# Regional Variation in Rates of IVF Treatment across Australia: A Population-based Study

**DOI:** 10.36469/9795

**Published:** 2017-03-23

**Authors:** Louise Rawlings, Pauline Ding, Stephen J. Robson

**Affiliations:** 1 Australian National University, Canberra, Australia

**Keywords:** assisted reproductive technology, ivf, clinical variation, socio-economic factors, access, health economics

## Abstract

**Background:** There is variation in uptake of in vitro fertilisation (IVF) between countries, and Australia has high incidence rates of IVF due to universal public funding. However, it remains unclear whether there is regional variation and, if present, what might cause this.

**Objectives:** We sought to determine whether regional variations in treatment rates existed and what might influence these.

**Methods:** The number of cycles of fresh IVF and intrauterine insemination (IUI) for women were obtained for the period 2011 until 2014 in two age groups (25 to 34 years and 35 to 44 years) to calculate incidence rates. Proxy indicators that might influence treatment affordability were: unemployment rates; average weekly total earnings; coverage of private health insurance; and, percentage of women in the highest socioeconomic quintile. Measures of accessibility considered were percentage of the population remote from urban areas and average state population density. Linear regressions were performed using log-transformed ratio of IVF and IUI incidence rates.

**Results:** Variations were found in IVF uptake between states with greater differences in older women. There was no significant association between IVF procedures and population density or geographic isolation. Economic factors were not associated with IVF uptake.

**Conclusion:** These findings suggest that factors such as physician preference, clinical practice guidelines, and cryopreservation protocols of ART units might explain the national variation in uptake of IVF.

## BACKGROUND

Assisted reproductive technology (ART) has advanced in scope and success rate over more than 30 years and has widespread availability in many countries now. In 2013 in Australia 4.4% of all births were the result of ART, the majority in women having their first child[1]: that rate is similar to other developed countries.[2] Despite the acceptance and uptake of ART there remains variation in its use internationally and it is recognised that many factors influence the rate of ART usage in different parts of the world.[3] While some influences such as public funding of *in vitro* fertilisation (IVF) treatment are relatively easy to study quantitatively other factors, in particular socio-religious and cultural influences, have not been extensively investigated. In addition, the rate of ART uptake is likely to vary according to physician preference, national or jurisdictional guidance regarding indications for treatment, and also the results of individual ART units and practices regarding cryopreservation.

In Australia, where both IVF and intrauterine insemination (IUI) receive public funding, there currently is no limit on the number of cycles that can be funded and no clinical eligibility criteria associated with public funding. Thus Australia fares well in terms of the affordability of ART for patients compared to many other countries, fostering a high proportion of IVF cycles that culminate in single embryo transfer (SET).[3] At present there are no national guidelines regarding clinical indications for the use of IVF in Australia, and de-identified data regarding live birth rates for non-donor cycles using fresh embryo transfers are published to allow comparision of the performance of ART clinics.[4] In view of the potential differences in indications, clinical practices, and outcomes for IVF we set out to determine whether there were associated differences in IVF uptake a regional level in Australia.

## METHODS

Because of universal health funding through Medicare Australia, all Australian citizens and permanent residents are eligible for a financial rebate for IVF procedures and intrauterine insemination (IUI) cycles. To ascertain the number of cycles of fresh IVF and IUI, we used data from the Medical Benefits Schedule (MBS) statistical database for the four-year period from January 2011 until December 2014. These data were consolidated into two age groups for women – 25 to 34 years of age (the 'younger age group'), and 35 to 44 years (the 'older age group') – for the largest five states in Australia (New South Wales, Victoria, Queensland, Western Australia, and South Australia). It is important to note that there is no difference in eligibility of women to receive funding from Medicare Australia between any state in Australia. To specify stimulated IVF cycles we used the MBS item number for oocyte retrieval (13212), and for IUI cycles we used the MBS item number for IUI (13203). To provide denominators for calculation of procedural incidence rates we obtained point estimates of the total female population of each age group in each of the five states from the Australian Bureau of Statistics (ABS) for each year of the study.

Because Medicare funds a proportion, but not all, of the cost of treatment for both IVF and IUI cycles most women will have to pay an out-of-pocket cost. There is thus a potential for affordability of IVF and IUI to vary according to socio-economic factors from two aspects: economic ability for IVF; and, geographical access. We considered four potential proxy indicators for the economic ability for IVF in each state as: (1) state-level unemployment rate; (2) average weekly total earning per person; (3) the proportion of the population with private health insurance; and, (4) the percentage of women aged 30 to 49 years in the top 20% of Socio-Economic Indexes for Areas (SEIFA).[5] As they would impact on affordability, we hypothesised that lower rates of unemployment, higher average weekly earnings, a higher proportion of the population with private health insurance and a higher percentage of the population in the top 20% SEIFA would be associated with a higher uptake of IVF procedures.

Since most ART units are located in capital cities or large regional centres, we also considered relative accessibility by using two factors: the percentage of the population residing outside of capital city and significant urban areas ('geographic isolation') and averaged each state population density per squarekilometre. We hypothesised that a higher percentage of the population living outside of capital city and significant urban areas and a lower population density to translate into lower uptake of IVF procedures as access would be more difficult. The socio-economic data were calculated from the relevant ABS datasets: ABS 6202.0 - *Labour Force, Australia*[6]; ABS 6302.0 - *Average Weekly Earnings, Australia*[7]; ABS 3101.0 - *Australian Demographic Statistics8*; ABS 3218.0 - *Regional Population Growth*[9]; The Private Health Insurance Administration Council annual coverage report[10]; and, a customised data set obtained from the ABS. The study received approval from the Human Research Ethics Committee of the Australian National University (protocol 2015/347).

**Statistical Analyses**

Data were extracted to Excel™ spreadsheets and statistical analysis was performed in GenStat and SPSS. Since there are no national guidelines regarding the indications for either IUI or IVF, we hypothesised that in some cases there would be a substitution of IUI cycles for IVF and took this into account in the analysis. The effect of each of the socio-economic factors was examined separately using linear regressions on the ratio of incident rates of oocyte retrievals and intrauterine insemination, indicating the possible substitution of IUI procedures over IVF. The analyses used log-transformed ratio data to satisfy the homogeneity of the variance assumption for linear regression.

## RESULTS

Our analysis was divided into two parts: firstly, to review the incidence rates of IVF and IUI in Australia from 2011 to 2014, separating women in the younger and older age groups. Secondly, we examined the effects of the socio-economic factors measuring affordability and remoteness/population density as measures of accessibility.

There were marked variations in the incidence rate of stimulated IVF cycles (oocyte retrievals per 1000 women per year) between the five states in both the younger (Figure 1) and older (Figure 2) age groups, with greater differences found in the older age group. The between-state variations in incidence rates of IUI cycles were even greater, but of similar magnitude in both the younger and older cohorts (Figures 3 and 4). Taking into account the possibility of a substitution of IUI for IVF in some women, Figure 5 demonstrates the trend of IVF over IUI at state level and the national level in both age groups: as expected the incidence rate of IVF was higher in the older age group in all five states. Across both the younger and older cohorts the highest uptake rates were in the states of Victoria and South Australia. Table 1 shows detailed age-stratified incidence rates of IVF and IUI. It was notable that, compared to the IVF incidence rates, uptake of IUI showed much greater variation between the states. Over the study period the incidence rates of both IVF and IUI remained relatively stable in other states, there was a notable increase in the IVF incidence rate in South Australia in 2013 (a 1.72 increase in the younger age group and a 3.38 increase in the older age group). South Australia had the lowest incidence rates of IUI compared to other states during the study period.

Results from linear regressions (Table 2) directly compare the association between the potentially influential socioeconomic factors and the uptake of IVF procedures. The directional relationships between the socioeconomic factors and the uptake of IVF were consistent in both age groups across the study period. With respect to geographical access to ART units there was no significant association in either age group between the incidence rate of IVF procedures and population density or geographic isolation ($p = 0.31$ for women in the younger age group, and $p = 0.12$ for women in the older age group). While the overall state population density was not significantly associated for younger women it was positively related to the uptake of IVF for older women.

In terms of the affordability for IVF most of the factors studied did not show a significant association with the uptake of IVF in either age group. Counterintuitively, in the younger age group a higher percentage of population holding private health insurance was significantly associated with a lower uptake of IVF procedures ($p = 0.043$). However, the negative relationship appeared to be driven by the high level of private health insurance coverage and low uptake of IVF in Western Australia.

| *in vitro* fertilisation (cycles per 1000 women) | | | | | | | |
|---|---|---|---|---|---|---|---|
| | | NSW | VIC | QLD | SA | WA | National |
| 25-34 | 2011 | 7.79 | 8.58 | 7.27 | 7.41 | 6.23 | 7.69 |
| | 2012 | 8.01 | 8.39 | 8.51 | 7.57 | 6.51 | 8.00 |
| | 2013 | 8.19 | 7.71 | 8.11 | 9.29 | 6.01 | 7.87 |
| | 2014 | 6.07 | 7.19 | 7.08 | 8.56 | 5.57 | 6.75 |
| 35-44 | 2011 | 14.45 | 15.97 | 9.73 | 9.98 | 10.32 | 13.56 |
| | 2012 | 14.59 | 17.69 | 10.85 | 9.89 | 9.60 | 13.72 |
| | 2013 | 15.57 | 16.72 | 10.77 | 13.27 | 9.90 | 14.02 |
| | 2014 | 15.46 | 17.31 | 11.21 | 10.47 | 10.64 | 14.13 |
| Intrauterine Insemination (cycles per 1000 women) | | | | | | | |
| | | NSW | VIC | QLD | SA | WA | National |
| 25-34 | 2011 | 2.26 | 2.07 | 6.83 | 1.08 | 3.97 | 3.23 |
| | 2012 | 2.53 | 2.09 | 6.26 | 1.94 | 3.85 | 3.28 |
| | 2013 | 2.25 | 1.74 | 5.60 | 2.26 | 3.18 | 2.92 |
| | 2014 | 2.27 | 1.55 | 4.48 | 1.48 | 3.13 | 2.57 |
| 35-44 | 2011 | 2.56 | 1.92 | 5.29 | 1.04 | 3.68 | 2.99 |
| | 2012 | 2.59 | 2.01 | 4.67 | 1.27 | 3.16 | 2.90 |
| | 2013 | 2.43 | 1.80 | 4.46 | 1.30 | 3.13 | 2.75 |
| | 2014 | 2.70 | 2.08 | 4.50 | 1.68 | 3.20 | 2.93 |

| | 25-34 | | | 35-44 | | |
|---|---|---|---|---|---|---|
| | estimate | s.e. | P values | estimate | s.e. | P values |
| Geographical isolation | -0.0444 | 0.0424 | 0.312 | -0.0694 | 0.0424 | 0.123 |
| Population density | 0.0268 | 0.0133 | 0.061 | 0.0352 | 0.0128 | 0.015* |
| Total earnings | -0.0345 | 0.0188 | 0.088 | -0.0338 | 0.0207 | 0.125 |
| Unemployment rates | 0.273 | 0.212 | 0.218 | 0.306 | 0.220 | 0.186 |
| Private health insurance | -0.0551 | 0.025 | 0.043* | -0.0507 | 0.0272 | 0.082 |
| top 20% SEIFA | 0.0237 | 0.0629 | 0.714 | 0.0103 | 0.0696 | 0.885 |

[* indicates the significance at 95% confidence level]

## CONCLUSIONS

Although definitions vary, and precise population-based estimates are difficult to obtain, it is likely that in developed countries the prevalence of 'infertility' is somewhere between 6.6 and 26.4%.[11] In Australia it has been estimated that approximately one couple in six have experienced a delay of greater than one year in achieving a planned pregnancy during their reproductive lives.[12] For this reason infertility has become a significant public health issue with an accompanying increase in demand for ART.[13]

This study suggests that there is considerable variation in the incidence rate of IVF within Australia, and that this variation is not completely explained by a substitution with non-IVF ART procedures such as IUI, or by socio-economic influences such as geographical isolation from ART units or socio-economic factors that could impact on affordability of treatment for couples. In Australia there is no over-arching national guidance regarding suitability or eligibility for IVF treatment, such as that published by NICE in the United Kingdom[14]: the choice of treatment is largely a matter of physician preference. Another factor that might be influential is the practice of embryo cryopreservation.

The absolute need for IVF treatment is obviously difficult to estimate, and this need will be dependent upon the pregnancy rate of treatment at a population level. However it has been estimated that at least 1500 IVF cycles per million people per year are needed to meet demand.[15] The uptake of IVF treatment does not correlate directly with clinical need, and there are marked differences in the incidence rate of IVF treatments between developed countries.[16] These differences have been attributed in large measure to the affordability of treatment, and specifically policies of public funding for ART.[17]

International comparative studies have revealed that, between countries, the average cost that patients pay for ART treatment relative to individual income is significantly associated with access to treatment.[18] Australia has been used as exemplar of supportive public funding of IVF treatment,[17] although it has been reported in other international studies that the number of IVF clinics per reproductive-age woman is an independent predictor of ART utilisation suggesting at least some degree of 'supplier-induced demand in the ART market.'[18]

In the absence of clear guidance as to which women or couples ought to have IVF treatment, and a policy of liberal access to funding of IVF cycles, the optimal rate of IVF uptake is difficult to measure. This is particularly so because of the relative imprecision of the definition of 'infertility'[19]: even with the use of a definition for infertility of 'failure to conceive after one year,' as many as 50% of couples would be expected to become pregnant without treatment.[20,21,22,23,24]

An important principle of public funding for health care in Australia is equity of access, but from the Government perspective considerations of cost-effectiveness are also relevant. There is evidence that decreasing affordability of treatment is associated with a greater rate of discontinuation of treatment by women in older age groups, possibly due to poorer prognosis.[18] The extent to which IVF treatment represents a valuable investment of public health resources has been subject to public debate in Australia.[25] Ideally, cost-effectiveness studies should take into account the age of the woman, the number of cycles required to pregnancy, comparator treatments, and potential complications such as multiple and preterm birth. Fortunately, the multiple birth rate for IVF-conceived pregnancies in Australia is less than 6% due to the high rate of SET which is almost 80%.[4] However it is recognised that SET will increase the number of embryo transfer cycles required. Yet despite the low rate of IVF-associated multiple birth in Australia there are increased odds for preterm birth and caesarean section (Box 1) both of which increase the cost both of birth and, for preterm birth, for longer-term care in childhood. Also, it has been noted that broader societal costs are underestimated commonly, including lost work productivity during treatment and the cost of counselling and support.[13]

| Outcome | IVF | non-IVF | OR | 95% CI | *p*-value* |
|---|---|---|---|---|---|
| *Preterm birth* | | | | | |
| 20 – 27 weeks | 1.62% | 0.75% | 2.44 | 2.11 - 2.81 | < 0.005 |
| 29 – 31 weeks | 1.68% | 0.64% | 2.95 | 2.55 – 3.40 | < 0.005 |
| 32 – 36 weeks | 14.3% | 5.9% | 2.75 | 2.62 – 2.90 | < 0.005 |
| *Caesarean section* | 50.1% | 31.6% | 2.18 | 2.11 – 2.26 | < 0.005 |

Data extracted from references [1] and [4].

Economies across the developed world are increasingly constrained fiscally and health expenditure sits within this environment. For example, in Australia health expenditure has increased from 6.5% of gross domestic product (GDP) in 1989-90 to 9.7% of GDP in 2013-14, with an increase in spending over that 25-year period from $50.3 billion to $154.6 billion in real terms.[26] Australian Government support for IVF and other ARTs must be viewed in this context, and Government funding of IVF raises questions for health economists.[27] Should limitations be placed on funding of treatment in situations where there is a poor prognosis, such as older women? In certain circumstances, should funding for IVF only be available when treatments such as weight loss and lifestyle modification have been unsuccessful? If fertility treatment is viewed as a 'market' then, in the absence of outside pressure, the market will commonly work in the interest of creating 'new business.' There may even be information asymmetry for patients seeking treatment to the point of market failure warranting government intervention.

At present Australia has a good record of achievement in ART and public funding of IVF has benefitted tens of thousands of families. However, it is important for all those involved in the provision of IVF services to ensure that treatment is used appropriately and performed to the highest standards possible to avoid perceptions of unwarranted variation.

## DISCLOSURE STATEMENT

The authors declare that there is no conflict of interest regarding the publication of this paper.

## Figures and Tables

**Figure:** Figure 1. Age-stratified incidence rate of in vitro fertilisation (IVF) cycles proceeding to oocyte retrieval in women aged 24 to 35 years in Australia (oocyte retrieval procedures per 1000 women per year), 2011 to 2014 inclusive

**Figure:** Figure 2. Age-stratified incidence rate of in vitro fertilisation (IVF) cycles proceeding to oocyte retrieval in women aged 35 to 44 years in Australia (oocyte retrieval procedures per 1000 women per year), 2011 to 2014 inclusive

**Figure:** Figure 3. Age-stratified incidence rate of intrauterine insemination (IUI) cycles in women aged 24 to 34 years in Australia (cycles per 1000 women per year), 2011 to 2014 inclusive

**Figure:** Figure 4. Age-stratified incidence rate of intrauterine insemination (IUI) cycles in women aged 35 to 44 years in Australia (cycles per 1000 women per year), 2011 to 2014 inclusive

**Figure:** Figure 5. Age-stratified ratio of IVF cycles versus intrauterine insemination IUI cycles in women (a) aged 25 to 34 years and (b) aged 35 to 44 years in Australia, 2011 to 2014 inclusive

**Figure:** Table 1. Age-stratified incidence rates of in vitro fertilisation cycles proceeding to oocyte retrieval and intrauterine insemination cycles (cycles per 1000 women per year) from 2011 to 2014 for women in two age groups: 25 to 34 years and 35 to 44 years

**Figure:** Table 2. Regression analysis results of effect of socio-economic factors for women in two age groups: 25 to 34 years and 35 to 44 years

**Figure:** Box 1. Proportions, odds ratios (OR) and 95% confidence intervals for preterm birth (stratified by gestation at birth) and caesarean delivery for IVF and non-IVF pregnancies in Australia in 2013. [*χ-square

